# Supplementary material for: A comprehensive transformer-based approach for high-accuracy gas adsorption predictions in metal-organic frameworks
Source: Nat Commun. 2024 Mar 1;15:1904. doi: 10.1038/s41467-024-46276-x (PMC10907743; doi:10.1038/s41467-024-46276-x)
Supplement: Supplementary file 1 — Supplementary Information [file 41467_2024_46276_MOESM1_ESM.pdf]

Supplementary Information

Supplementary Tables

Supplementary Table 1. Prediction performance of multi-gas adsorption uptake under varied working conditions. Dataset divided into three splits (train:valid:test = 8:1:1) by material structure. R^2 is the coefficient of determination.

Data Source	Gas	Temperature	Pressure	Property	Data Point	Uni-MOF
hMOF_MOFX-DB	CO_2, N_2, CH_4, Kr, Xe	273K, 298K	0.01-10Pa	adsorption uptake (mol kg^{-1})	2,477,494	0.983
CoRE_MOFX-DB	Ar, N_2	77K, 87K	$1-10^5$Pa	adsorption uptake (cm^3_{STP} g^{-1})	464,824	0.916
CoRE_MAP-DB	CH_4, CO_2, Ar, Kr, Xe, O_2, He	150-300K	1Pa-3bar	adsorption uptake (cm^3_{STP} g^{-1})	99,200	0.834

Supplementary Table 2. Descriptors of Gas Molecules.

Gas	Critical Temperature (K)	Critical Pressure (MPa)	Acentric Factor	Molecular Mass	Melting point (K)	Boiling point (K)
CH_4	190.8	4.60	0.011	16.0	90.7	112
CO_2	304.2	7.38	0.239	44.0	217	195
Ar	151.2	4.87	0.001	39.9	83.8	87.3
Kr	209.3	5.53	0.005	83.8	116	120
Xe	289.7	5.84	0.008	131	161	165
O_2	154.6	5.05	0.025	32.0	54.4	90.2
He	2.200	0.23	-0.390	4.00	0.95	4.22
N_2	126.2	3.40	0.039	28.0	63.1	77.4

Supplementary Table 3. Outliers information from CoRE_MOFX_DB database. Here OMS means Open Metal Site.

MOF materials	Metal Types	PLD (Å)	LCD (Å)	Metal Site Energy Parameter ε (K)	Has OMS	OMS types	Is Disordered
NIBJAK_clean	Cu	17.29	32.05	2.52	Yes	Cu	No
XAHQAA_clean	Cu	21.24	22.55	2.52	Yes	Cu	No
APUPEH_clean	Cu	14.03	24.88	2.52	Yes	Cu	No
FEBXIV_clean	Cu	15.15	16.82	2.52	Yes	Cu	No
LELDOX_clean	Cu	6.35	13.15	2.52	Yes	Cu	Yes
XONLES01_clean	Cu	13.54	14.83	2.52	Yes	Cu	No
REZXUQ_clean	Cr	13.92	15.62	7.55	Yes	Cr	No
ja5111317_ja5111317_si_003_clean	Zr	11.28	31.48	34.7	No	N/A	Yes

Supplementary Table 4. Percentage of MOFs with open metal sited in outliers with greatest absolute prediction error from CoRE_MOFX_DB database. Here OMS means Open Metal Site.

Outliers	Percentage of MOFs with OMS
Top 10	80%
Top 100	74%

Supplementary Table 5. Predictive performance of Uni-MOF in three databases (with pre-training of MOF and COF database). R^2 is the coefficient of determination. *RMSE* represents Root Mean Square Error, *MAE* represents Mean Absolute Error.

Database	R^2 (higher is better ↑)	RMSE (lower is better ↓)	MAE (lower is better ↓)
hMOF_MOFX_DB	0.983	0.274 ($mol\ kg^{-1}$)	0.14 ($mol\ kg^{-1}$)
CoRE_MOFX_DB	0.916	83.83 ($cm^3_{STP}\ g^{-1}$)	42.2 ($cm^3_{STP}\ g^{-1}$)
CoRE_MAP_DB	0.834	13.76 ($cm^3_{STP}\ g^{-1}$)	3.48 ($cm^3_{STP}\ g^{-1}$)

Supplementary Table 6. Predictive performance of Uni-MOF in three databases (with pre-training of MOF only). R^2 is the coefficient of determination. *RMSE* represents Root Mean Square Error. *MAE* represents Mean Absolute Error.

Database	R^2 (higher is better ↑)	RMSE (lower is better ↓)	MAE (lower is better ↓)
hMOF_MOFX_DB	0.970	0.331 ($mol\ kg^{-1}$)	0.17 ($mol\ kg^{-1}$)
CoRE_MOFX_DB	0.874	84.63 ($cm^3_{STP}\ g^{-1}$)	42.5 ($cm^3_{STP}\ g^{-1}$)
CoRE_MAP_DB	0.742	21.48 ($cm^3_{STP}\ g^{-1}$)	6.61 ($cm^3_{STP}\ g^{-1}$)

Supplementary Table 7. Summary information of top 100 outlier in CoRE_MOFX_DB database. OMS means Open Metal Site. MOF represents Metal-Organic Framework.

metal	MOFs	MOFs_with_OMS	Metal Sites	MOFs_with_OMS(%)
Cu	31	31	51	100.00%
Zn	23	12	38	52.17%
Fe	9	5	15	55.56%
Zr	8	3	13	37.50%
Cd	6	3	9	50.00%
Co	5	4	10	80.00%
Ni	4	4	5	100.00%
Mn	4	1	5	25.00%
Cr	3	2	3	66.67%
Tb	3	2	4	66.67%
Dy	2	2	6	100.00%
Na	2	2	2	100.00%
Si	2	1	3	50.00%
Mg	2	2	2	100.00%
Hf	2	1	4	50.00%
Eu	1	1	1	100.00%
Ce	1	1	1	100.00%
K	1	1	7	100.00%
Sm	1	1	1	100.00%
In	1	0	1	0.00%
U	1	1	2	100.00%
V	1	0	1	0.00%
Nd	1	0	2	0.00%

Supplementary Table 8. Experimental Adsorption Uptake Data. MOF represents Metal-Organic Framework.

MOF	gas	temperature (K)	pressure (Pa)	predictive value (cm^3_{STP} g^{-1})	adsorption uptake (cm^3_{STP} g^{-1})	Reference
Zn2(bdc)2(dabco)	CH_4	393	1	-0.11	–	this work
Zn2(bdc)2(dabco)	CH_4	393	10	-0.01	–	this work
Zn2(bdc)2(dabco)	CH_4	393	100	0.10	–	this work
Zn2(bdc)2(dabco)	CH_4	393	1000	1.23	–	this work
Zn2(bdc)2(dabco)	CH_4	393	1×10^4	11.7	–	this work
Zn2(bdc)2(dabco)	CH_4	393	5×10^4	54.0	–	this work
Zn2(bdc)2(dabco)	CH_4	393	1×10^5	103	–	this work
Zn2(bdc)2(dabco)	CH_4	393	2×10^5	158	–	this work
Zn2(bdc)2(dabco)	CH_4	393	3×10^5	172	–	this work
Zn2(bdc)2(dabco)	CH_4	393	3.5×10^6	–	175	[1]
MIL-101	CH_4	373	1	-0.08	–	this work
MIL-101	CH_4	373	10	-0.05	–	this work
MIL-101	CH_4	373	100	0.04	–	this work
MIL-101	CH_4	373	1000	0.54	–	this work
MIL-101	CH_4	373	1×10^4	5.68	–	this work
MIL-101	CH_4	373	5×10^4	21.0	–	this work
MIL-101	CH_4	373	1×10^5	35.7	–	this work
MIL-101	CH_4	373	2×10^5	68.8	–	this work
MIL-101	CH_4	373	3×10^5	85.3	–	this work
MIL-101	CH_4	373	3.5×10^6	–	135	[1]
Mg-dobdc	CO_2	298	1	0.09	–	this work
Mg-dobdc	CO_2	298	10	0.05	–	this work
Mg-dobdc	CO_2	298	100	0.07	–	this work
Mg-dobdc	CO_2	298	1000	0.44	–	this work
Mg-dobdc	CO_2	298	1×10^4	3.13	–	this work
Mg-dobdc	CO_2	298	5×10^4	14.1	–	this work
Mg-dobdc	CO_2	298	1×10^5	35.5	–	this work
Mg-dobdc	CO_2	298	2×10^5	64.1	–	this work
Mg-dobdc	CO_2	298	3×10^5	82.3	–	this work
Mg-dobdc	CO_2	298	1×10^4	–	112	[2]
Mg-dobdc	CO_2	298	1×10^5	–	179	[2]
Mg-dobdc	CH_4	298	1	0.02	–	this work
Mg-dobdc	CH_4	298	10	0.03	–	this work
Mg-dobdc	CH_4	298	100	0.03	–	this work
Mg-dobdc	CH_4	298	1000	0.17	–	this work
Mg-dobdc	CH_4	298	1×10^4	1.62	–	this work
Mg-dobdc	CH_4	298	5×10^4	8.02	–	this work
Mg-dobdc	CH_4	298	1×10^5	17.7	–	this work
Mg-dobdc	CH_4	298	2×10^5	36.9	–	this work
Mg-dobdc	CH_4	298	3×10^5	52.3	–	this work
Mg-dobdc	CH_4	298	1×10^5	–	24.9	[2]

Supplementary Table 9. Experimental Adsorption Uptake Data. MOF represents Metal-Organic Framework.

MOF	gas	temperature (K)	pressure (Pa)	predictive value (cm^3_{STP} g^{-1})	adsorption uptake (cm^3_{STP} g^{-1})	Reference
MOF-5	CH_4	298	1	0.04	–	this work
MOF-5	CH_4	298	10	0.01	–	this work
MOF-5	CH_4	298	100	0.04	–	this work
MOF-5	CH_4	298	1000	0.11	–	this work
MOF-5	CH_4	298	1×10^4	0.94	–	this work
MOF-5	CH_4	298	5×10^4	4.41	–	this work
MOF-5	CH_4	298	1×10^5	10.4	–	this work
MOF-5	CH_4	298	2×10^5	18.9	–	this work
MOF-5	CH_4	298	3×10^5	28.0	–	this work
MOF-5	CH_4	298	1×10^5	–	3.23	[3]
MOF-5	CH_4	298	3×10^5	–	4.39	[3]
MOF-5	CH_4	298	5×10^5	–	9.05	[3]
MOF-5	CH_4	298	9×10^5	–	16.2	[3]
MOF-5	CH_4	298	1.2×10^6	–	17.8	[3]
MOF-5	CH_4	298	1.5×10^6	–	19.6	[3]
MOF-5	CH_4	298	1.86×10^6	–	21.4	[3]
MOF-5	CH_4	298	2.38×10^6	–	22.7	[3]
MOF-5	CH_4	298	2.74×10^6	–	23.0	[3]
MOF-5	CH_4	298	3×10^6	–	23.3	[3]
MOF-177	CH_4	298	1	-0.01	–	this work
MOF-177	CH_4	298	10	0.01	–	this work
MOF-177	CH_4	298	100	0.02	–	this work
MOF-177	CH_4	298	1000	0.09	–	this work
MOF-177	CH_4	298	1×10^4	0.60	–	this work
MOF-177	CH_4	298	5×10^4	1.93	–	this work
MOF-177	CH_4	298	1×10^5	3.08	–	this work
MOF-177	CH_4	298	2×10^5	4.27	–	this work
MOF-177	CH_4	298	3×10^5	4.79	–	this work
MOF-177	CH_4	298	1×10^5	–	4.17	[3]
MOF-177	CH_4	298	4×10^5	–	7.37	[3]
MOF-177	CH_4	298	7.5×10^5	–	14.6	[3]
MOF-177	CH_4	298	1.02×10^6	–	22.2	[3]
MOF-177	CH_4	298	1.3×10^6	–	25.3	[3]
MOF-177	CH_4	298	1.6×10^6	–	27.5	[3]
MOF-177	CH_4	298	2.02×10^6	–	29.1	[3]
MOF-177	CH_4	298	2.21×10^6	–	29.8	[3]
MOF-177	CH_4	298	2.55×10^6	–	31.1	[3]
MOF-177	CH_4	298	2.72×10^6	–	31.4	[3]
MOF-177	CH_4	298	3×10^6	–	32.6	[3]

Supplementary Table 10. Experimental Adsorption Uptake Data. MOF represents Metal-Organic Framework.

MOF	gas	temperature (K)	pressure (Pa)	predictive value (cm^3_{STP} g^{-1})	adsorption uptake (cm^3_{STP} g^{-1})	Reference
MOF-5	CO_2	296	1	-0.04	–	this work
MOF-5	CO_2	296	10	0.01	–	this work
MOF-5	CO_2	296	100	0.09	–	this work
MOF-5	CO_2	296	1000	0.28	–	this work
MOF-5	CO_2	296	1×10^4	1.48	–	this work
MOF-5	CO_2	296	5×10^4	7.23	–	this work
MOF-5	CO_2	296	1×10^5	14.7	–	this work
MOF-5	CO_2	296	2×10^5	32.5	–	this work
MOF-5	CO_2	296	3×10^5	49.3	–	this work
MOF-5	CO_2	296	1.01×10^5	–	47.1	[4]
MOF-5	CO_2	298	1	0.05	–	this work
MOF-5	CO_2	298	10	0.09	–	this work
MOF-5	CO_2	298	100	0.07	–	this work
MOF-5	CO_2	298	1000	0.33	–	this work
MOF-5	CO_2	298	1×10^4	1.75	–	this work
MOF-5	CO_2	298	5×10^4	7.45	–	this work
MOF-5	CO_2	298	1×10^5	16.8	–	this work
MOF-5	CO_2	298	2×10^5	31.3	–	this work
MOF-5	CO_2	298	3×10^5	47.5	–	this work
MOF-5	CO_2	298	1.01×10^5	–	43.0	[5]
MOF-5	CO_2	298	1.01×10^5	–	41.5	[6]
MOF-5	CO_2	298	1.01×10^5	–	25.3	[7]
MOF-5	CO_2	298	1.01×10^5	–	25.1	[8]

Supplementary Table 11. Prediction of adsorption uptake with the random division inito three dataset (train, validation and test dataset with ratio of 5:1:1) according to adsorbate gases. R^2 is the coefficient of determination.

Data Source	Gas in test set	R^2
CoRE_MAP_DB	Ar	0.617
CoRE_MAP_DB	CH_4	0.465
CoRE_MAP_DB	CO_2	0.358
CoRE_MAP_DB	Kr	0.851
CoRE_MAP_DB	He	0.449
CoRE_MAP_DB	O_2	0.370
CoRE_MAP_DB	Xe	0.405

Supplementary Table 12. Properties of gas molecules.

Gas	kinetic diameter (pm)	LJ parameter ε (K)	LJ parameter σ (Å)
CH_4	330	159	3.72
CO_2	380	258	3.71
Ar	340	93.1	3.45
Kr	360	111	3.69
Xe	396	167	3.92
O_2	346	113	3.43
He	260	28.2	2.10

Supplementary Table 13. Prediction of adsorption uptake with the division inito two dataset (train and test dataset with ratio of 6:1) according to adsorbate gases. R^2 is the coefficient of determination.

Data Source	Gas in test set	R^2
CoRE_MAP_DB	Ar	0.801
CoRE_MAP_DB	CH_4	0.771
CoRE_MAP_DB	CO_2	0.506
CoRE_MAP_DB	Kr	0.881
CoRE_MAP_DB	He	0.451
CoRE_MAP_DB	O_2	0.408
CoRE_MAP_DB	Xe	0.492

Supplementary Table 14. Predictive performance of Uni-MOF in COF adsorption database. R^2 is the coefficient of determination. *RMSE* represents Root Mean Square Error. *MAE* represents Mean Absolute Error.

Database	R^2 (higher is better ↑)	RMSE (cm^3_{STP} g^{-1}) (lower is better ↓)	MAE (cm^3_{STP} g^{-1}) (lower is better ↓)
COF_DB	0.758	4.280	1.55

Supplementary Table 15. Comparison of Model Performance of argon adsorption uptake $(\mathrm{cm}^3_{\mathrm{STP}}\ \mathrm{g}^{-1})$ on CoRE_MOFX-DB. Dataset divided into three splits (train:valid:test = 8:1:1), Uni-MOF$_{single}$ denotes to Uni-MOF model under single working condition's training data, while Uni-MOF$_{mixed}$ indices the mixed mutli-gas absorption model under varied working conditions training in the whole hMOF dataset, that is one vs all performance. The numbers in bold represent cases in which the predictions of the mixed model outperform those of the single model. R^2 is the coefficient of determination.

Temperature (K)	Pressure (Pa)	Data Points	R^2 Uni-MOF$_{single}$	R^2 Uni-MOF$_{mixed}$
87K	0.001	12,018	0.681	0.537
87K	0.01	12,018	0.677	0.626
87K	0.1	12,018	0.662	**0.716**
87K	1	12,018	0.671	**0.688**
87K	10	12,018	0.725	**0.762**
87K	100	12,018	0.793	**0.805**
87K	1,000	12,018	0.848	**0.870**
87K	2,000	12,018	0.839	**0.893**
87K	4,000	12,018	0.852	**0.873**
87K	6,000	12,018	0.834	**0.906**
87K	8,000	12,018	0.899	0.890
87K	10,000	12,018	0.911	0.903
87K	12,000	12,018	0.903	**0.912**
87K	14,000	12,018	0.923	0.917
87K	16,000	12,018	0.915	**0.930**
87K	18,000	12,018	0.813	**0.930**
87K	20,000	12,018	0.916	**0.930**
87K	24,000	12,018	0.920	**0.929**
87K	28,000	12,018	0.910	**0.930**
87K	30,000	12,018	0.904	**0.927**
87K	32,000	12,018	0.912	**0.929**
87K	36,000	12,018	0.892	**0.928**
87K	40,000	12,018	0.905	**0.928**
87K	50,000	12,018	0.910	0.900
87K	60,000	12,018	0.882	**0.898**
87K	70,000	12,018	0.845	**0.898**
87K	80,000	12,018	0.847	**0.895**
87K	90,000	12,018	0.715	**0.895**
87K	100,000	12,018	0.684	**0.896**

Supplementary Table 16. Comparison of Model Performance of nitrogen adsorption uptake (cm^3_{STP} g^{-1}) on CoRE_MOFX-DB. Dataset divided into three splits (train:valid:test = 8:1:1), Uni-MOF$_{single}$ denotes to Uni-MOF model under single working condition's training data, while Uni-MOF$_{mixed}$ indices the mixed mutli-gas absorption model under varied working conditions training in the whole hMOF dataset, that is one vs all performance. The numbers in bold represent cases in which the predictions of the mixed model outperform those of the single model. R^2 is the coefficient of determination.

Temperature (K)	Pressure (Pa)	Data Points	R^2 Uni-MOF$_{single}$	R^2 Uni-MOF$_{mixed}$
77K	1	8,388	0.690	**0.782**
77K	4	8,388	0.734	**0.795**
77K	10	3,630	0.753	**0.789**
77K	18	8,356	0.770	**0.828**
77K	75	8,270	0.852	0.827
77K	80	3,630	0.714	**0.787**
77K	316	3,596	0.709	**0.851**
77K	200	8,173	0.876	**0.879**
77K	500	3,555	0.792	**0.863**
77K	1,000	3,504	0.880	0.853
77K	1,334	8,002	0.779	**0.916**
77K	1,500	3,452	0.752	**0.858**
77K	2,000	3,389	0.831	**0.855**
77K	5,000	3,316	0.860	0.842
77K	5,623	7,828	0.863	**0.934**
77K	20,000	3,247	0.833	**0.911**
77K	23,714	7,659	0.878	**0.942**
77K	40,000	3,182	0.876	**0.879**
77K	60,000	3,130	0.759	**0.871**
77K	80,000	3,084	0.731	**0.868**
77K	99,900	3,045	0.576	**0.869**
77K	100,000	7,478	0.911	0.862

Supplementary Table 17. Comparison of Model Performance on hMOF absorption uptake (mol kg^{-1}). Dataset divided into three splits (train:valid:test = 8:1:1), Uni-MOF$_{single}$ denotes to Uni-MOF model under single working condition's training data, while Uni-MOF$_{mixed}$ indices the mixed mutli-gas absorption model under varied working conditions training in the whole hMOF dataset, that is one vs all performance. The numbers in bold represent cases in which the predictions of the mixed model outperform those of the single model. R^2 is the coefficient of determination.

Gas	Temperature (K)	Pressure (bar)	Data Points	R^2 Uni-MOF$_{single}$	R^2 Uni-MOF$_{mixed}$
CO_2	298	0.01	137,652	0.839	**0.859**
CO_2	298	0.05	137,652	0.898	**0.916**
CO_2	298	0.1	137,652	0.915	**0.928**
CO_2	298	0.5	137,652	0.941	**0.952**
CO_2	298	2.5	137,652	0.963	0.956
Kr	273	1	137,410	0.909	**0.961**
Kr	273	5	137,652	0.947	**0.969**
Kr	273	10	137,652	0.932	**0.939**
N_2	298	0.09	137,652	0.847	0.802
CH_4	298	0.05	137,652	0.862	**0.865**
CH_4	298	0.5	137,652	0.929	**0.938**
CH_4	298	0.9	137,652	0.938	**0.948**
CH_4	298	2.5	137,652	0.954	**0.966**
CH_4	298	4.5	137,652	0.961	**0.974**
Xe	273	1	137,652	0.952	**0.961**
Xe	273	5	137,652	0.970	**0.976**
Xe	273	10	137,652	0.959	**0.962**
N_2	298	0.9	137,652	0.909	**0.920**

Supplementary Table 18. Prediction of intrinsic structural feature for CoRE MOFs and hMOFs. Dataset divided into three splits (train:valid:test = 8:1:1). R^2 is the coefficient of determination.

Data Source	Property	Data Point	R^2 Uni-MOF$_{single}$
CoRE MOF	PLD (Å)	12,018	0.880
CoRE MOF	LCD (Å)	12,018	0.916
CoRE MOF	void fraction	12,018	0.936
CoRE MOF	pore volume (cm^3 g^{-1})	12,018	0.947
hMOF	PLD (Å)	137,953	0.991
hMOF	LCD (Å)	137,953	0.991
hMOF	void fraction	137,953	0.999
hMOF	pore volume (cm^3 g^{-1})	137,953	0.999

Supplementary Table 19. Force field parameters of noble gases.

Gas	σ (Å)	ε (K)
Argon	3.41	119.5
Krypton	3.66	165.2
Xenon	3.97	229.8

Supplementary Figures

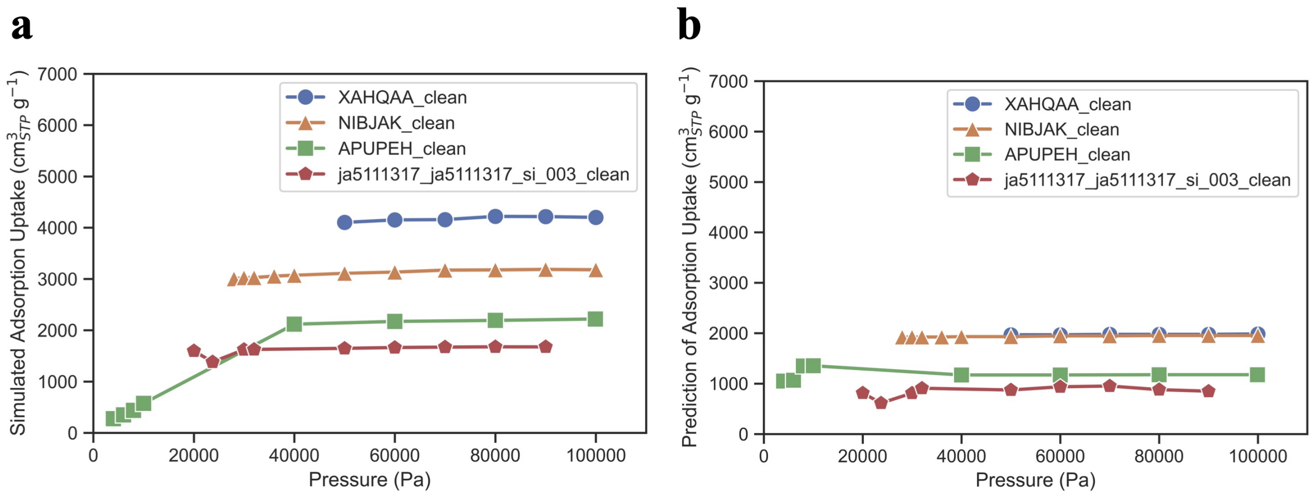

Supplementary Fig. 1. (a) simulated and (b) predicted adsorption uptake illustration of outliers in CoRE_MOFX_DB database. Source data are provided as a Source Data file.

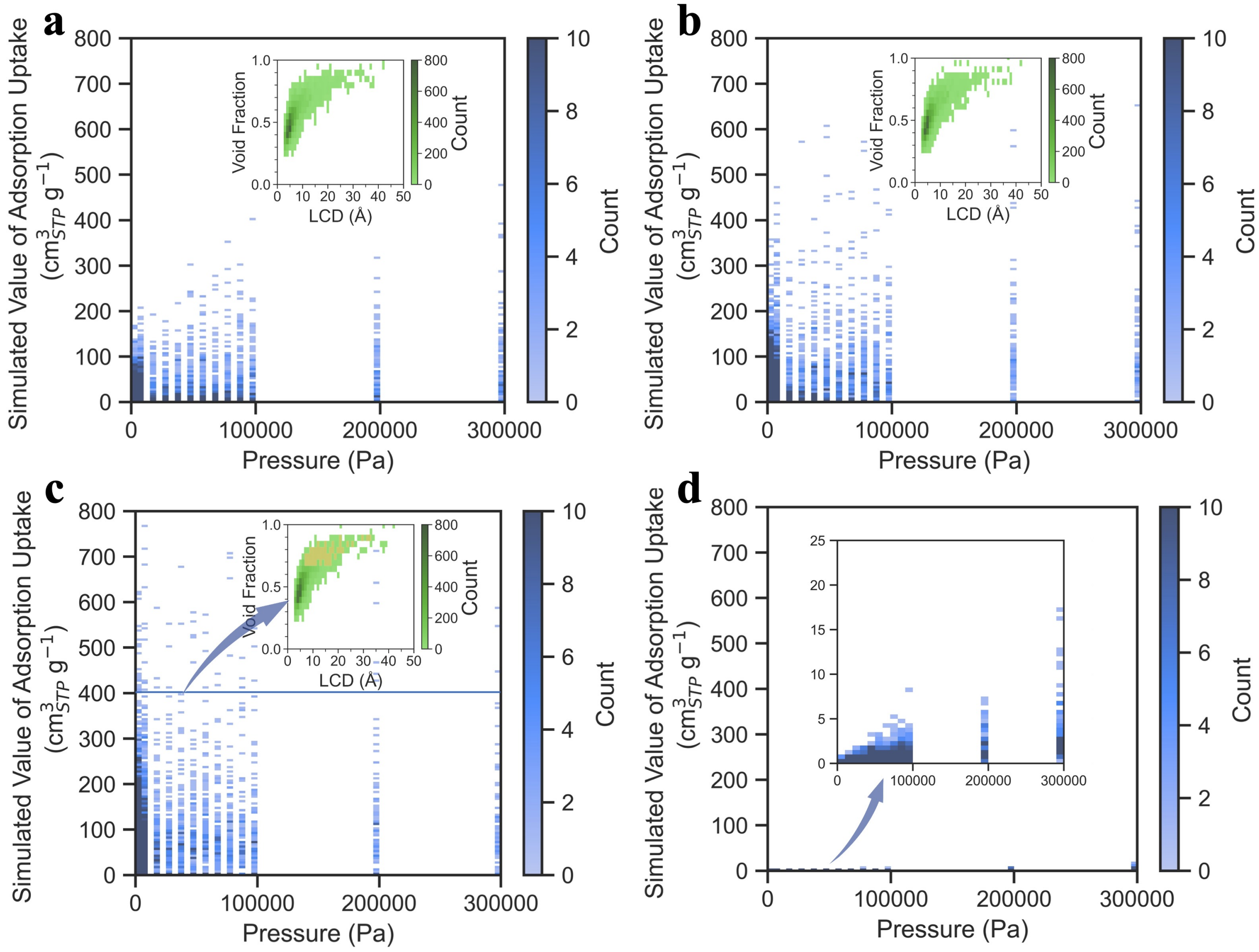

Supplementary Fig. 2. Distribution of pressure and simulated adsorption uptake of **a** Ar, **b** Kr, **c** Xe and **d** He. Source data are provided as a Source Data file.

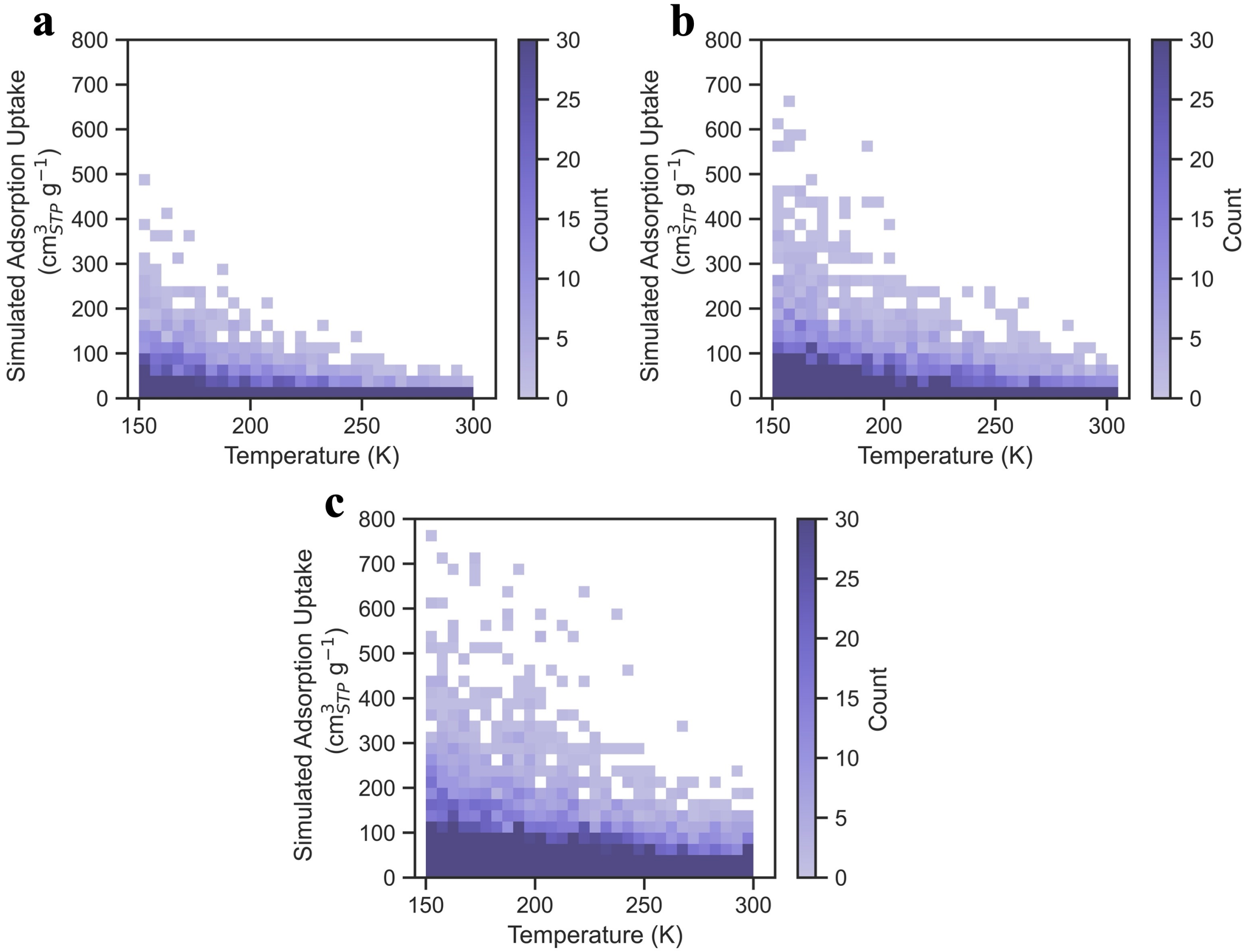

Supplementary Fig. 3. Distribution of temperature and simulated adsorption uptake of **a** Ar, **b** Kr and **c** Xe. Source data are provided as a Source Data file.

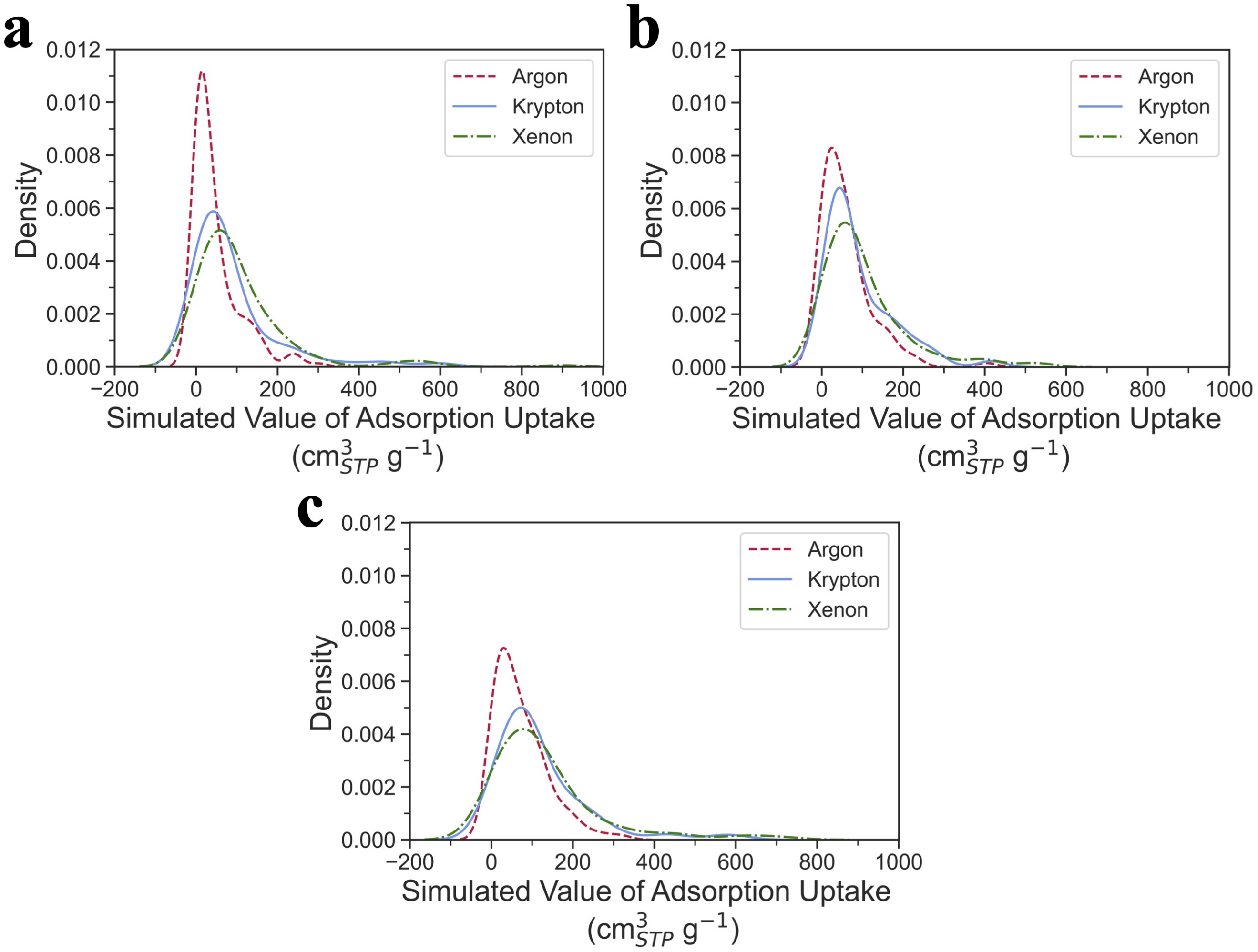

Supplementary Fig. 4. Distribution of simulated adsorption uptake of Ar, Kr and Xe under pressure of **a** 50 kPa, **b** 100 kPa and **c** 200 kPa. Source data are provided as a Source Data file.

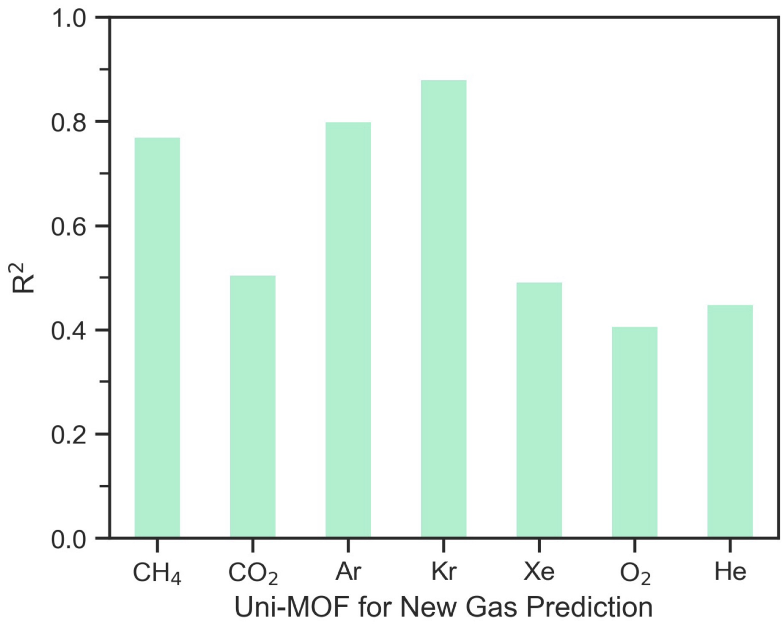

Supplementary Fig. 5. Prediction of adsorption uptake with the division inito two dataset (train and test dataset with ratio of 6:1) according to adsorbate gases.

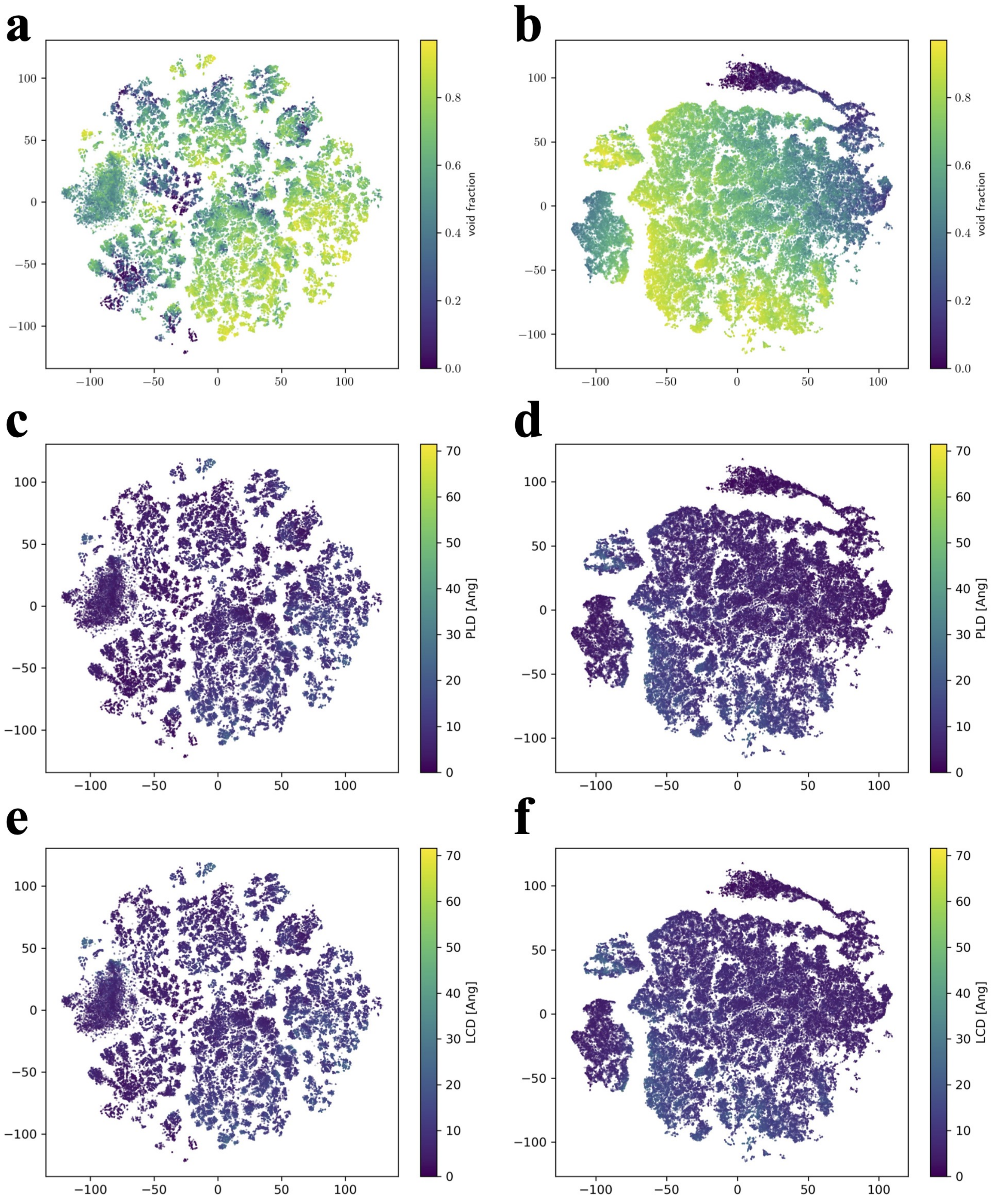

Supplementary Fig. 6. Visualization of structural representations of MOF in the hMOF and CoRE_MOF datasets, the low-dimensional embeddings are computed by t-SNE. The t-SNE, *i.e.*, t-distributed stochastic neighbor embedding method, is an effective approach for dimensionality reduction and visualization of high-dimensional matrices. The representations retrieved after **a, c, e** pre-training and **b, d, f** fine-tuning versus the other properties. **a** and **b** illustrate the representations *vs* void fraction for hMOF and CoRE_MOF combined. **c,d** and **e,f** illustrate the representations *vs* PLD and LCD in Å for hMOF and CoRE_MOF combined.

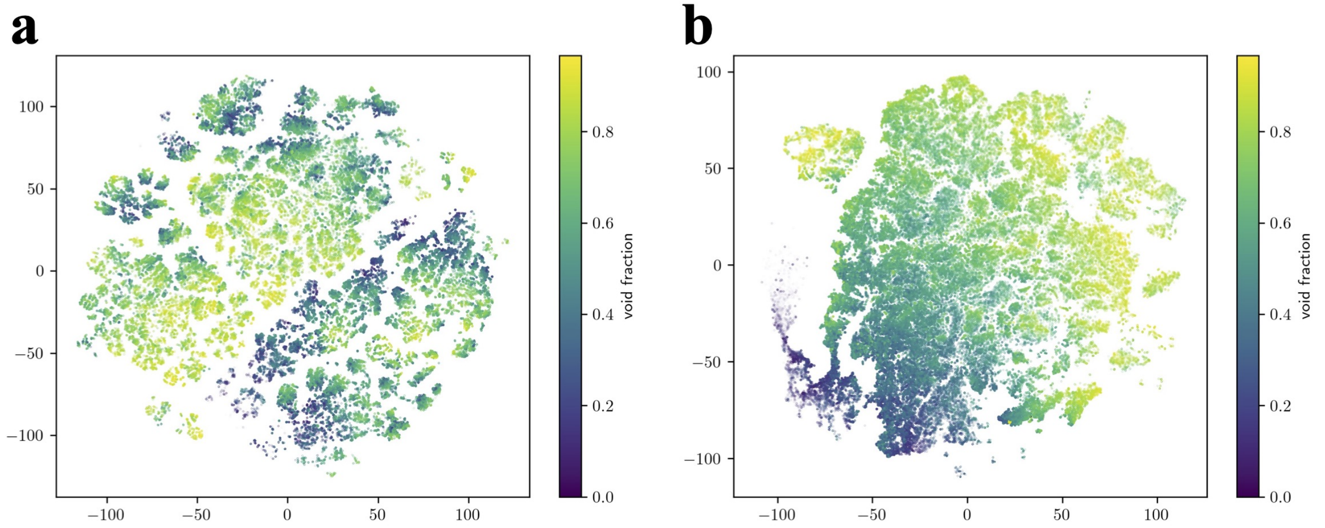

Supplementary Fig. 7. Visualization of structural representations of MOF in the hMOF dataset, the low-dimensional embeddings are computed by t-SNE. The t-SNE, *i.e.*, t-distributed stochastic neighbor embedding method, is an effective approach for dimensionality reduction and visualization of high-dimensional matrices. The representations retrieved after **a** pre-training and **b** fine-tuning versus the void fraction.

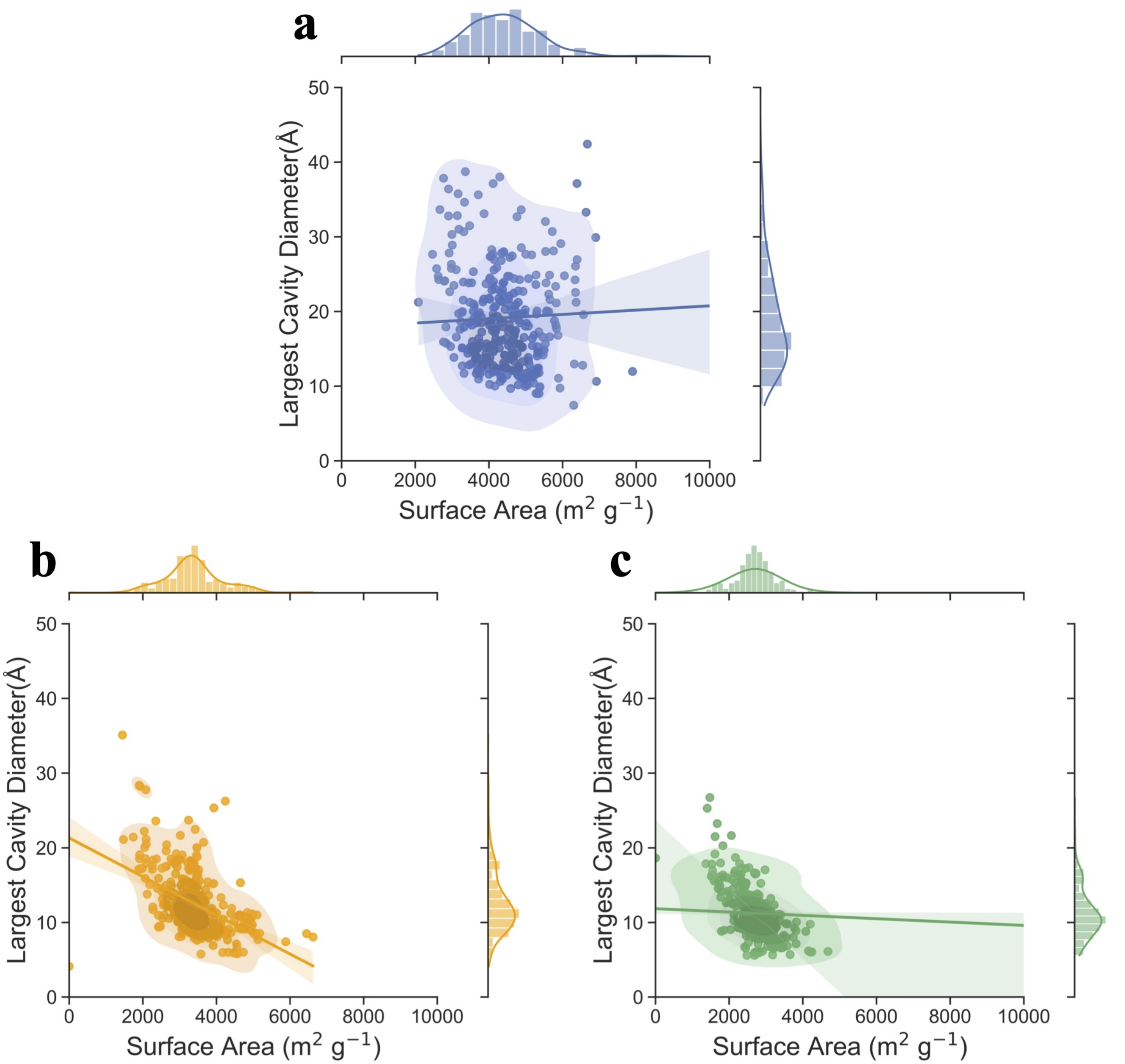

Supplementary Fig. 8. Distribution of surface area and LCD (Largest Cavity Diameter) for **a** 1-tier, **b** 2-tier, **c** 3-tier within top 10% of MOFs (Metal-Organic Frameworks) according to their adsorption performance on argon.

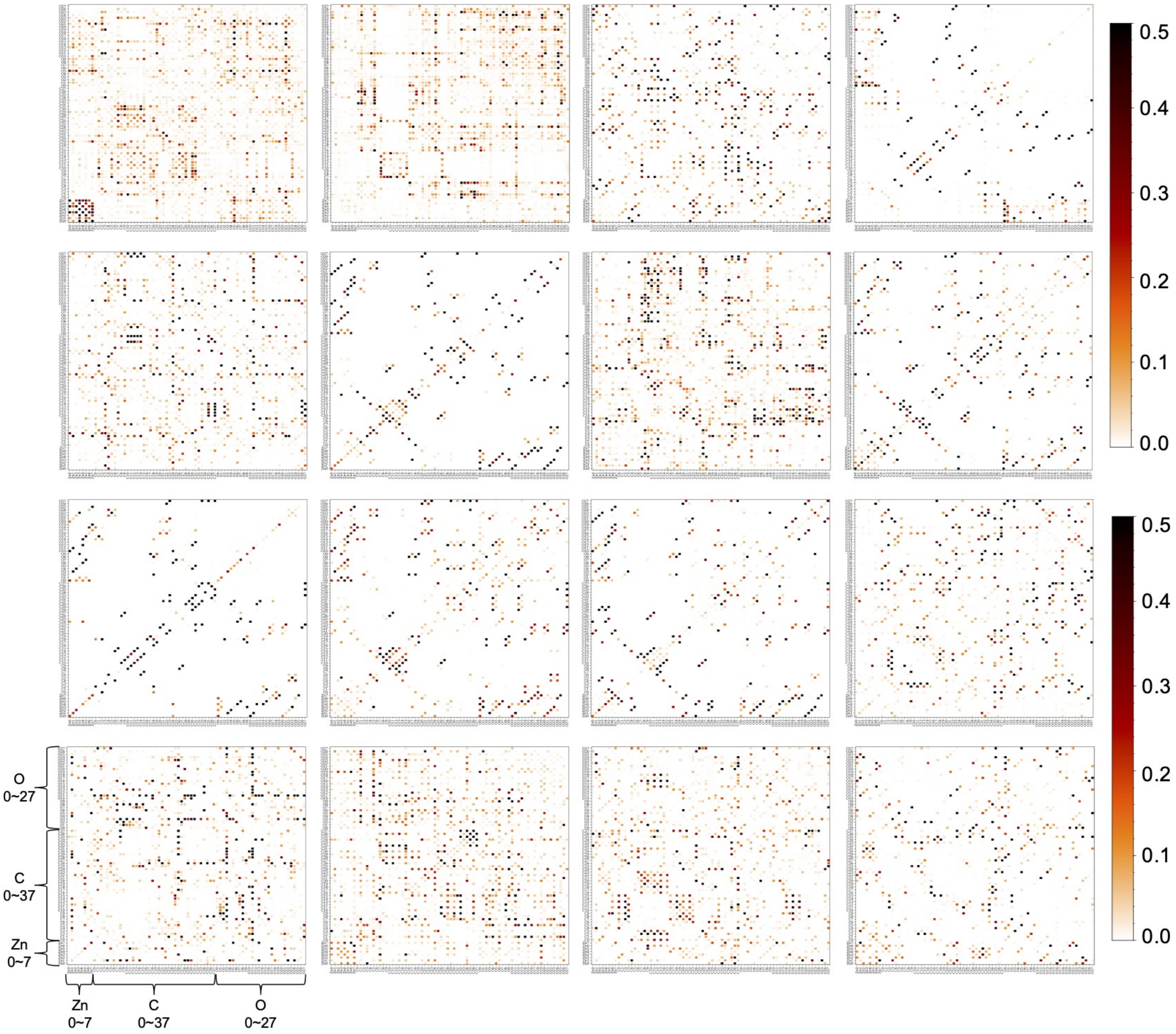

Supplementary Fig. 9. Heat map of atomic interactions in hMOF-5004238 from Head 1-16.

Supplementary Notes

Loss function for regression

The loss function in machine learning is a measure of how well a machine learning model predicts the expected outcome. The loss function will take two inputs: the output value of our model and the value of the ground truth. Here we introduce the coefficient of determination (R^2) and other 2 most common loss functions for Machine Learning regression. For instance, a data set has N values marked $[y_1, ..., y_N]$, each associated with a predicted value $[f_1, ..., f_N]$.

Coefficient of determination, R^2

$$\bar{y} = \frac{1}{N}\sum_{i=1}^{N} y_i \qquad \text{(Supplementary Equation (1))}$$

where $\bar{y}$ is the mean of the observed data.

$$SS_{res} = \sum_{i=1}^{N}(y_i - f_i)^2 = \sum_{i=1}^{N} e_i^2 \qquad \text{(Supplementary Equation (2))}$$

where SS_{res} represents the sum of squares of residuals, namely the residual sum of squares.

$$SS_{tot} = \sum_{i=1}^{N}(y_i - \bar{y})^2 \qquad \text{(Supplementary Equation (3))}$$

where SS_{tot} means the total sum of squares.

$$R^2 = 1 - \frac{SS_{res}}{SS_{tot}} \qquad \text{(Supplementary Equation (4))}$$

where R^2 is the coefficient of determination. In the ideal case where the predicted values exactly match the observed values, $SS_{res} = 0$ and $R^2 = 1$. A baseline model, which always predicts the mean value $\bar{y}$, will have $R^2 = 0$. A worse model than the baseline will have a negative R^2.

Root mean square error (RMSE)

$$RMSE = \sqrt{\frac{1}{N}\sum_{i=1}^{N}(y_i - f_i)^2} \qquad \text{(Supplementary Equation (5))}$$

RMSE is able to ensure that our trained model has no outlier predictions with huge errors due to the squared part in the function.

Mean absolute error (MAE)

$$MAE = \frac{1}{N}\sum_{i=1}^{N}|y_i - f_i| \qquad \text{(Supplementary Equation (6))}$$

Since we are taking absolute values in MAE, all errors will be weighted on the same linear scale. So, unlike MSE, where we don't put too much weight on outliers, and our loss function provides a generic, even way to measure the model's performance.

Supplementary References

1. Senkovska, I. & Kaskel, S. High pressure methane adsorption in the metal-organic frameworks cu3 (btc) 2, zn2 (bdc) 2dabco, and cr3f (h2o) 2o (bdc) 3. *Microporous Mesoporous Mater.* **112**, 108–115 (2008).
2. Nugent, P. *et al.* Porous materials with optimal adsorption thermodynamics and kinetics for co2 separation. *Nature* **495**, 80–84 (2013).
3. Nath, K., Ahmed, A., Siegel, D. J. & Matzger, A. J. Microscale determination of binary gas adsorption isotherms in mofs. *J. Am. Chem. Soc.* **144**, 20939–20946 (2022).
4. Zhao, Z., Li, Z. & Lin, Y. Adsorption and diffusion of carbon dioxide on metal- organic framework (mof-5). *Ind. & Eng. Chem. Res.* **48**, 10015–10020 (2009).
5. Walton, K. S. *et al.* Understanding inflections and steps in carbon dioxide adsorption isotherms in metal-organic frameworks. *J. Am. Chem. Soc.* **130**, 406–407 (2008).
6. Yaghi, O. M. & Millward, A. R. Metal-organic frameworks with exceptionally high capacity for storage of carbon dioxide at room-temperature (2010). US Patent 7,799,120.
7. Son, W.-J., Kim, J., Kim, J. & Ahn, W.-S. Sonochemical synthesis of mof-5. *Chem. Commun.* 6336–6338 (2008).
8. Choi, J.-S., Son, W.-J., Kim, J. & Ahn, W.-S. Metal–organic framework mof-5 prepared by microwave heating: Factors to be considered. *Microporous Mesoporous Mater.* **116**, 727–731 (2008).
