# Supplementary material for: A comprehensive transformer-based approach for high-accuracy gas adsorption predictions in metal-organic frameworks
Source: Nat Commun. 2024 Mar 1;15:1904. doi: 10.1038/s41467-024-46276-x (PMC10907743; doi:10.1038/s41467-024-46276-x)
Supplement: Supplementary file 3 — Reporting Summary [file 41467_2024_46276_MOESM3_ESM.pdf]

nature portfolio

Corresponding author(s): NCOMMS-23-22528C

Last updated by author(s): Jan 16, 2024

Reporting Summary

Nature Portfolio wishes to improve the reproducibility of the work that we publish. This form provides structure for consistency and transparency in reporting. For further information on Nature Portfolio policies, see our Editorial Policies and the Editorial Policy Checklist.

Statistics

For all statistical analyses, confirm that the following items are present in the figure legend, table legend, main text, or Methods section.

n/a	Confirmed	
☐	☒	The exact sample size (*n*) for each experimental group/condition, given as a discrete number and unit of measurement
☐	☒	A statement on whether measurements were taken from distinct samples or whether the same sample was measured repeatedly
☒	☐	The statistical test(s) used AND whether they are one- or two-sided<br>*Only common tests should be described solely by name; describe more complex techniques in the Methods section.*
☒	☐	A description of all covariates tested
☒	☐	A description of any assumptions or corrections, such as tests of normality and adjustment for multiple comparisons
☐	☒	A full description of the statistical parameters including central tendency (e.g. means) or other basic estimates (e.g. regression coefficient) AND variation (e.g. standard deviation) or associated estimates of uncertainty (e.g. confidence intervals)
☒	☐	For null hypothesis testing, the test statistic (e.g. *F*, *t*, *r*) with confidence intervals, effect sizes, degrees of freedom and *P* value noted<br>*Give P values as exact values whenever suitable.*
☒	☐	For Bayesian analysis, information on the choice of priors and Markov chain Monte Carlo settings
☐	☒	For hierarchical and complex designs, identification of the appropriate level for tests and full reporting of outcomes
☒	☐	Estimates of effect sizes (e.g. Cohen's *d*, Pearson's *r*), indicating how they were calculated

Our web collection on statistics for biologists contains articles on many of the points above.

Software and code

Policy information about availability of computer code

Data collection	There is a wealth of existing MOF/COF databases, including computer-synthesized databases of hMOFs, ToBaCCo (Topologically Based Crystal Constructor) MOFs, and experimental-level databases of CoRE (Computation-Ready Experimental) MOFs, CoRE COFs and CCDC (The Cambridge Crystallographic Data Centre), etc. One integrated database online is MOFXDB, where more than 168,000 MOF/COF structures are available. Additionally, we used the ToBaCCo.3.0 program to generate over 300,000 MOF structures. For the downstream task, i.e., gas adsorption uptake by MOFs, we collected data from online sources such as MOFXDB. Another dataset was generated by Grand Canonical Monte Carlo(GCMC) simulations on RASPA software.
Data analysis	We use the pymatgen (Python Materials Genomics), a robust and open-source Python library, to derive useful material properties from raw crystallographic structural data and conduct comprehensive materials analysis. To further investigate the effect of material structure on gas adsorption, Zeo++, a software package for crystalline porous materials analysis, was used to perform an analysis of the structure and topology of the material geometry. Python data visualization libraries such as seaborn and matplotlib were used for informative statistical graphics. Three-dimensional structures are drawn using the web service Bohrium.

For manuscripts utilizing custom algorithms or software that are central to the research but not yet described in published literature, software must be made available to editors and reviewers. We strongly encourage code deposition in a community repository (e.g. GitHub). See the Nature Portfolio guidelines for submitting code & software for further information.

Data

Policy information about availability of data

All manuscripts must include a data availability statement. This statement should provide the following information, where applicable:

- Accession codes, unique identifiers, or web links for publicly available datasets
- A description of any restrictions on data availability
- For clinical datasets or third party data, please ensure that the statement adheres to our policy

All the data and code about the Uni-MOF model is available at https://github.com/dptech-corp/Uni-MOF.

Research involving human participants, their data, or biological material

Policy information about studies with human participants or human data. See also policy information about sex, gender (identity/presentation), and sexual orientation and race, ethnicity and racism.

Reporting on sex and gender	There was no human participants or human data in our work.
Reporting on race, ethnicity, or other socially relevant groupings	There was no human participants or human data in our work.
Population characteristics	There was no human participants or human data in our work.
Recruitment	There was no human participants or human data in our work.
Ethics oversight	There was no human participants or human data in our work.

Note that full information on the approval of the study protocol must also be provided in the manuscript.

Field-specific reporting

Please select the one below that is the best fit for your research. If you are not sure, read the appropriate sections before making your selection.

☐ Life sciences ☐ Behavioural & social sciences ☒ Ecological, evolutionary & environmental sciences

For a reference copy of the document with all sections, see nature.com/documents/nr-reporting-summary-flat.pdf

Ecological, evolutionary & environmental sciences study design

All studies must disclose on these points even when the disclosure is negative.

Study description	We propose Uni-MOF, an innovative framework for large-scale, three-dimensional MOF representation learning, designed for universal multi-gas prediction. Different databases have different units (mol/kg and cm3/g). To prevent data bias and ensure that the test set remained unseen by the model, we divided the data set into three different data sets (train, valid and test) with the ratio of 8:1:1 according to the MOF structure instead of randomly splitting. Results in this work are reproducible.
Research sample	The data in our work have no relation to organisms, our work is to build a model for gas adsorption prediction. There is a wealth of existing MOF/COF databases, including computer-synthesized databases of hMOFs, ToBaCCo (Topologically Based Crystal Constructor) MOFs, and experimental-level databases of CoRE (Computation-Ready Experimental) MOFs and COFs, CCDC (The Cambridge Crystallographic Data Centre), etc. For the downstream task, i.e., gas adsorption uptake by MOFs, we collected data from online sources such as MOFXDB, and we collected another dataset by Grand Canonical Monte Carlo(GCMC) simulations on RASPA software.
Sampling strategy	To prevent data bias and ensure that the test set remained unseen by the model, we divided the data set into three different data sets (train, valid and test) with the ratio of 8:1:1 according to the MOF structure instead of randomly splitting, that is, there is no identical material between the three datasets.
Data collection	There is a wealth of existing MOF/COF databases, including computer-synthesized databases of hMOFs, ToBaCCo (Topologically Based Crystal Constructor) MOFs, and experimental-level databases of CoRE (Computation-Ready Experimental) MOFs and COFs, CCDC (The Cambridge Crystallographic Data Centre), etc. One integrated database online is MOFXDB, where more than 168,000 MOF/COF structures are available. Additionally, we used the ToBaCCo.3.0 program to generate over 300,000 MOF structures. For the downstream task, i.e., gas adsorption uptake by MOFs, we collected data from online sources such as MOFXDB, and we collected another dataset by Grand Canonical Monte Carlo(GCMC) simulations on RASPA software. Jingqi Wang and Jiapeng Liu collected and generated the databases.
Timing and spatial scale	The data were collected from July 2022 to September 2022, and it took us more than half a year to build the model and write the paper.
Data exclusions	No data was excluded.

Reproducibility	Since our work is about Machine Learning in material science, we share our code and data on GitHub, and our results are reproducible.
Randomization	For Machine Learning model, we do divide data. In our work, to prevent data bias and ensure that the test set remained unseen by the model, we divided the data set into three different data sets (train, valid and test) with the ratio of 8:1:1 according to the MOF structure instead of randomly splitting, that is, there is no identical material between the three datasets.
Blinding	Blinding was not relevant to our study, because in our work, we proposed Uni-MOF, an innovative framework for large-scale, three-dimensional MOF representation learning, designed for universal multi-gas prediction. We collected and generated extensive database thus to imporve the model performance. We also analyzed the predicted performance for test set of database. Thus, blinding was not relevant to our study.

Did the study involve field work? ☐ Yes ☒ No

Reporting for specific materials, systems and methods

We require information from authors about some types of materials, experimental systems and methods used in many studies. Here, indicate whether each material, system or method listed is relevant to your study. If you are not sure if a list item applies to your research, read the appropriate section before selecting a response.

Materials & experimental systems

n/a	Involved in the study
☒	☐ Antibodies
☒	☐ Eukaryotic cell lines
☒	☐ Palaeontology and archaeology
☒	☐ Animals and other organisms
☒	☐ Clinical data
☒	☐ Dual use research of concern
☒	☐ Plants

Methods

n/a	Involved in the study
☒	☐ ChIP-seq
☒	☐ Flow cytometry
☒	☐ MRI-based neuroimaging
